# Effect of trehalose on heart functions in rats model after myocardial infarction: assessment of novel intraventricular pressure and heart rate variability

**DOI:** 10.3389/fcvm.2023.1182628

**Published:** 2023-06-21

**Authors:** Ahmed Farag, Ahmed S. Mandour, Masahiro Kaneda, Ahmed Elfadadny, Asmaa Elhaieg, Kazumi Shimada, Ryou Tanaka

**Affiliations:** ^1^ Department of Veterinary Surgery, Faculty of Veterinary Medicine, Tokyo University of Agriculture and Technology, Fuchu, Japan; ^2^ Department of Surgery, Anesthesiology, and Radiology, Faculty of Veterinary Medicine, Zagazig University, Zagazig, Egypt; ^3^ Department of Animal Medicine (Internal Medicine), Faculty of Veterinary Medicine, Suez Canal University, Ismailia, Egypt; ^4^ Laboratory of Veterinary Anatomy, Division of Animal Life Science, Tokyo University of Agriculture and Technology, Tokyo, Japan; ^5^ Department of Animal Internal Medicine, Faculty of Veterinary Medicine, Damanhur University, Damanhur El-Beheira, Egypt

**Keywords:** myocardial infarction, trehalose, rat, intraventricular pressure gradients, heart rate variability

## Abstract

**Background:**

Myocardial infarctions remain a leading cause of global deaths. Developing novel drugs to target cardiac remodeling after myocardial injury is challenging. There is an increasing interest in exploring natural cardioprotective agents and non-invasive tools like intraventricular pressure gradients (IVPG) and heart rate variability (HRV) analysis in myocardial infarctions. Trehalose (TRE), a natural disaccharide, shows promise in treating atherosclerosis, myocardial infarction, and neurodegenerative disorders.

**Objectives:**

The objective of this study was to investigate the effectiveness of TRE in improving cardiac functions measured by IVPG and HRV and reducing myocardial remodeling following myocardial infarction in rat model.

**Methods:**

Rats were divided into three groups: sham, myocardial infarction (MI), and trehalose-treated MI (TRE) groups. The animals in the MI and TRE groups underwent permanent ligation of the left anterior descending artery. The TRE group received 2% trehalose in their drinking water for four weeks after the surgery. At the end of the experiment, heart function was assessed using conventional echocardiography, novel color M-mode echocardiography for IVPG evaluation, and HRV analysis. After euthanasia, gross image scoring, histopathology, immunohistochemistry, and quantitative real-time PCR were performed to evaluate inflammatory reactions, oxidative stress, and apoptosis.

**Results:**

The MI group exhibited significantly lower values in multiple IVPG parameters. In contrast, TRE administration showed an ameliorative effect on IVPG changes, with results comparable to the sham group. Additionally, TRE improved HRV parameters, mitigated morphological changes induced by myocardial infarction, reduced histological alterations in wall mass, and suppressed inflammatory reactions within the infarcted heart tissues. Furthermore, TRE demonstrated antioxidant, anti-apoptotic and anti-fibrotic properties.

**Conclusion:**

The investigation into the effect of trehalose on a myocardial infarction rat model has yielded promising outcomes, as evidenced by improvements observed through conventional echocardiography, histological analysis, and immunohistochemical analysis. While minor trends were noticed in IVPG and HRV measurements. However, our findings offer valuable insights and demonstrate a correlation between IVPG, HRV, and other traditional markers of echo assessment in the myocardial infarction vs. sham groups. This alignment suggests the potential of IVPG and HRV as additional indicators for future research in this field.

## 1. Introduction

Myocardial infarction (MI) is a major type of ischemic heart disease and is defined by an imbalance between ischemia and cardiac muscle cell death (1, 2). Despite noteworthy progress over the last 10 years, acute myocardial infarction is still the most extreme form of coronary artery disease, impacting almost 7 million individuals globally and responsible for more than 4 million deaths every year in Europe and Northern Asia (3, 4). Development of effective new cardioprotective agents that can limit the drawbacks of MI or remodeling, or the pathology of the heart in affected patients, in addition to novel non-invasive tools with the ability for early detection of heart dysfunction, is currently a major research concern.

For instance, trehalose, a disaccharide of glucose, has demonstrated potential advantages for cardiovascular health (5). It has the ability to reduce cardiomyocyte death *in vitro* in response to energy stress (6). The activation of autophagy by consuming trehalose orally significantly reduces cardiac remodeling, dysfunction, and heart failure in a mouse model of chronic MI (7). Additionally, in rats with ischemic cardiomyopathy undergoing surgical ventricular reconstruction, trehalose intake also decreased the recurrence of LV remodeling and altered autophagy markers (8).

Echocardiography is a common diagnostic tool in cardiovascular research trials and clinical settings. However, conventional echocardiography still has limitations in accurately evaluating diastolic functions, so cardiologists often rely on invasive catheterization to understand diastolic heart function, despite its drawbacks. Therefore, there is an increasing interest in noninvasive diagnostic tools. Currently, Color M-mode echocardiography (CMME) can be used to assess intraventricular pressure gradients in a noninvasive way (IVPG). The interventricular pressure gradient (IVPG) is the measurement of the difference in pressure between various points in the left ventricle at the beginning of diastole when the apical pressure is lower than the basal pressure (9). As compared to intrusive catheterization, IVPG evaluated by CMME is thought of as a precise assessment of diastolic function and has the plus of being reproducible (10–13).

Another new technique is heart rate variability (HRV), which gauges the variation in a heartbeat and has been shown to contain essential details about the regulation of the cardiovascular system. Particularly, the short-term behavior of HRV is widely used to analyze the impact of the autonomic nervous system on the heart in multiple situations (14, 15). It has been extensively studied how HRV indices correlate to the prognosis of some pathophysiological conditions, such as myocardial infarction and heart failure (HF), as they can be effective non-invasive biomarkers of cardiac risk (16–18). The aforementioned novel diagnostic techniques have been utilized in many research trials to assess heart function under heart failure or pharmacologically modulated heart function models (19, 20).

In recent years, numerous novel strategies, such as tissue engineering and regenerative nanomedicine applications, have been employed to repair injured cardiac tissue. However, myocardial damage following myocardial infarction is still challenging to recover from (21).

The potential beneficial effects of trehalose on acute myocardial infarction have not yet been fully studied. In this study, we hypothesized that trehalose could be a potentially useful compound for the treatment of acute ischemia remodeling, which can be detected by the early changes in IVPG and HRV. Therefore, we will investigate the effects of TRE postinfarction administration in the rat model and analyze its effect functionally via conventional echocardiography, IVPG, electrocardiogram (ECG) and HRV. Additionally, we will evaluate the histological changes and inflammatory cytokines expressions, as well as its antioxidant and anti-apoptotic effects in the created models.

## 2. Materials and methods

### 2.1. Animals and ethical approval

Twenty-four Sprague Dawley rats, ranging in age from 12 to 16 weeks and weighing between 350 and 400 grams, were used in the experiment. The experiment was conducted while abiding by the Guide for the Care and Use of Laboratory Animals and was authorized by Tokyo University of Agriculture and Technology's Institutional Animal Care and Use Committee (Approval No. R04-185). Rats had open access to food and water and were kept in an environment with a temperature of 20°C, with 12 h of light and 12 h of darkness reinforcing one another in an alternating pattern. Permanent ligation (PL) and ischemia-reperfusion (IR) procedures are valuable tools for investigating and replicating myocardial infarction. For examining post-MI tissue responses, a larger and more severe injury is preferable, as it allows for the examination of significant differences between damaged and healthy tissue. A less severe injury, such as the one caused by IR, may obscure otherwise significant differences. Therefore, for general research that compares injured and sham animals, the PL method may be more appropriate.

### 2.2. Experimental design

The rats were divided into three groups (*n* = 8): (1) Sham group; (2) Myocardial Infarction (MI) group, which was subjected to MI surgery by permanent left anterior descending coronary artery (LAD) ligation technique; and (3) Trehalose-treated group (MI + TRE), which was subjected to MI surgery at day 0 followed by drinking a 2% solution of trehalose (Trehalose dihydrate, FUJIFILM Wako Pure Chemical Corporation, Japan) in the drinking water (7, 22). All rats were kept under suitable environmental conditions and housed for four weeks.

### 2.3. Surgical induction of MI

Rats were anesthetized with a combination of medetomidine hydrochloride (Domitor, Orion Pharma Animal Health, Helsinki, Finland), midazolam (Dormicum, Astellas Pharma Inc., Tokyo, Japan), and butorphanol (Vetorphale, Meiji Seika Pharma Co., Ltd.) at a dose rate of 0.3, 5.0, and 5.0 mg/kg body weight, respectively. This combination was freshly prepared and mixed with sterile saline to form a stock solution. The rats were subcutaneously injected with 0.5 ml of the anesthetic mixture per 100 g of body weight. Once the loss of body righting reflex was noted, they were intubated using a 16-gauge IV catheter and maintained with isoflurane (Isoflurane, Pfizer Inc., New York, USA) at a concentration of 1.0%, and then placed in a supine position on a temperature-controlled pad to maintain core temperature of 35.5°C. After the surgery was completed, atipamezole (Antisedan, Orion Pharma Animal Health) was administered at a dose rate of 1.0 mg/kg for recovery (23).

A small cut between the third and fourth intercostal spaces was then made to carry out a left-sided thoracotomy. A blunt-ended retractor was used to open the incision away from the lung to prevent it from collapsing. A cut was made in the pericardial sac to access the heart 8 mm from the origin; the left anterior descending artery was tied off with a 6-0 polypropylene suture and secured with three knots (24). The blanching and discoloration of the left ventricle's anterior wall and the enlargement of the left atrium indicated successful ligation (25). Postoperative care was performed after recovery, including infection control with gentamicin (Nacalai Tesque Co., Ltd., Tokyo, Japan) (2.4 × 10^4^/kg/IP/days) for 3 days (26) and pain control with carprofen (Rimadyl, Zoetis Japan K.K., Tokyo, Japan) (5 mg/kg subcutaneously) with a pre-surgical one dose followed by two post-surgery doses (27).

### 2.4. Conventional echocardiography

Conventional echocardiography, CMME, ECG, and HRV were performed consecutively on the same individual rat on the same day. A 12 MHz ProSound F75 ultrasound system with a joint ECG (Hitachi-Aloka Medical Ltd., Tokyo, Japan) was implemented. The American Society of Echocardiography's (ASE) guidelines were followed for performing the echocardiography (28, 29). MMB was used to anesthetize all the animals for an easier and more practical examination. Through a two-dimensional right parasternal short-axis view of the left ventricle (LV) at the papillary muscles level, an M-mode imaging was achieved. The LV was manually measured by the same individual using the ASE-endorsed leading-edge technique, which has been confirmed to be suitable for utilization with the rat MI model (30, 31).

The left ventricular internal diameter during diastole (LVIDd), systole (LVIDs), and posterior wall's diameter during diastole (LVPWd) and systole (LVPWs), as well as the interventricular septal thickness in diastole (IVSd) and systole (IVSs), were documented from the apical four-chamber view. From the same view, the ejection fraction (EF%) and fractional shortening (FS%) along with the trans-mitral inflow indices, which included the early (E) and late (A) velocities and the E/A ratio through pulsed-wave (PW) and tissue Doppler (TD) imaging were also documented (32).

$$\begin{array}{rl} & \mathrm{Average}\;E/e^{\prime }\;\mathrm{was}\;\mathrm{calculated}\;\mathrm{as}\;\mathrm{follow}:\;E/e^{\prime }\\ & =(E/e^{\prime }\;\mathrm{lateral}+E/e^{\prime }\;\mathrm{septal})/2 \end{array}$$

$$\begin{array}{rl} & \mathrm{Average}\;\mathrm{Em}/\mathrm{Am}\;\mathrm{was}\;\mathrm{calculated}\;\mathrm{as}\;\mathrm{follows}:\;\mathrm{Em}/\mathrm{Am}\\ & =(\mathrm{Em}/\mathrm{Am}\;\mathrm{lateral}+\mathrm{Em}/\mathrm{Am}\;\mathrm{septal})/2. \end{array}$$

### 2.5. Color M-mode echocardiography for IVPG

Proper machine settings followed the previously published article in our laboratory (33). The IVPG was evaluated using the CMME. To ensure precise tracing of the CMME, the ultrasound machine was adjusted with a sweep speed of 300 mm/s and a color base shift of −64 to improve the Nyquist limitation. First, the flow path from the left atrium to the LV apex over the mitral valve was refined, followed by adjusting the left apical 4-chamber view. Subsequently, the M-mode was enabled to trace the inflow. Color M-mode images were collected and stored for later review in MATLAB (The MathWorks, Natick, MA, USA) (32).

A custom code created in MATLAB was utilized for analyzing the CMME images, incorporating the following image processing algorithm: (∂P))/(∂s)=−ρ⋅((∂v)/(∂t)+v⋅(∂v)/(∂s)).

**Figure 1:**
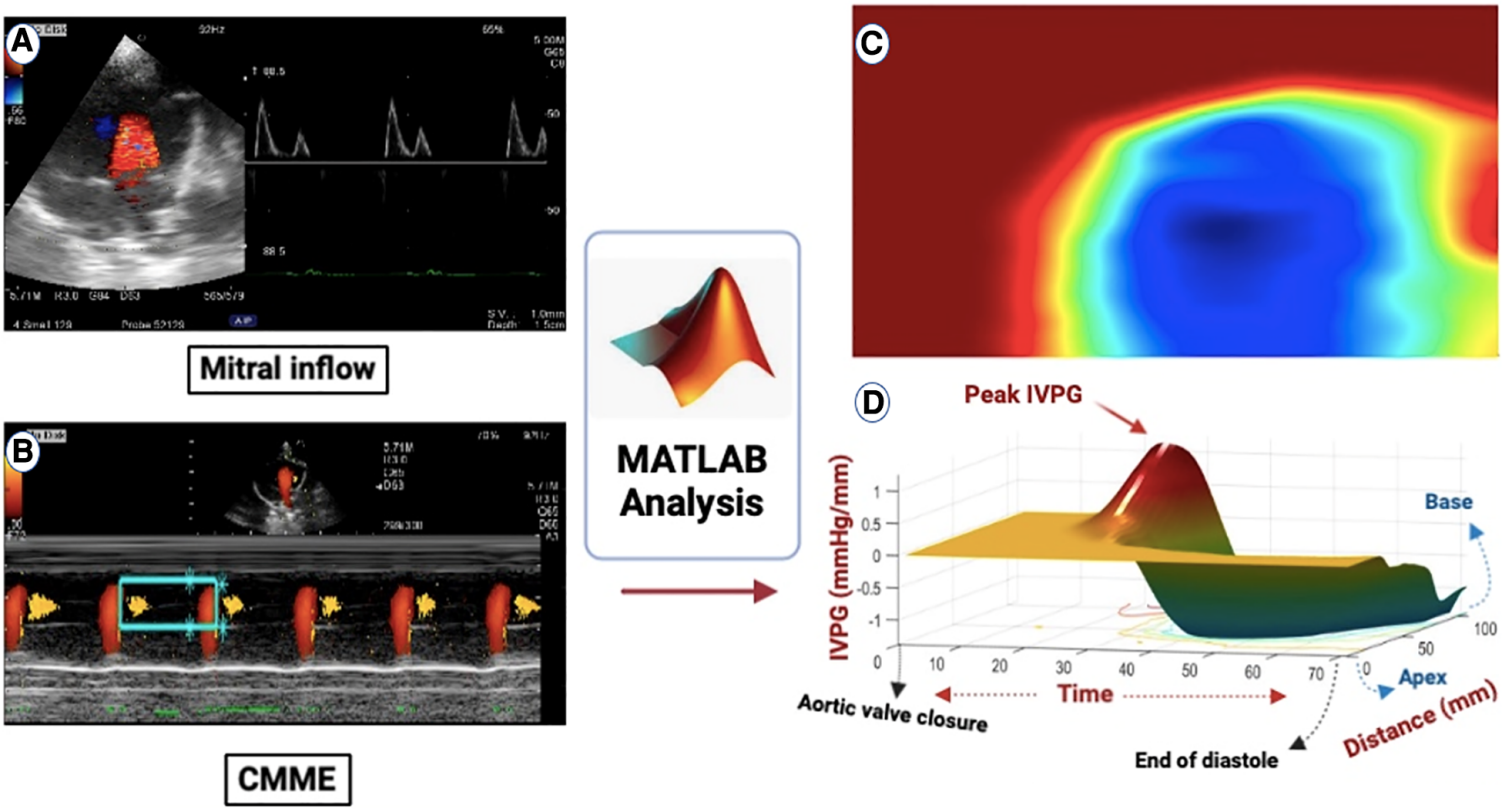
(**A**) All groups had the mitral inflow evaluated using pulsed-wave Doppler echocardiography from the left apical four-chamber view. (**B**) Apical four-chamber view CMME was employed to trace the path of the inflow from the left atrium (LA) to the apex of the left ventricle (LV) with the cursor parallel to the mitral inflow. (**C**) MATLAB software was used for further processing of the photos for IVPD and IVPG calculation. (**D**) CMME analysis with MATLAB software generated 3D temporal and spatial profiles of the IVPG based on Euler's equation over a certain portion of the systolic duration starting from the total closure of the aortic valve to the end of diastole. The z-axis represents the distance (mm) from the apex to the mitral valve (base).

**Figure 2:**
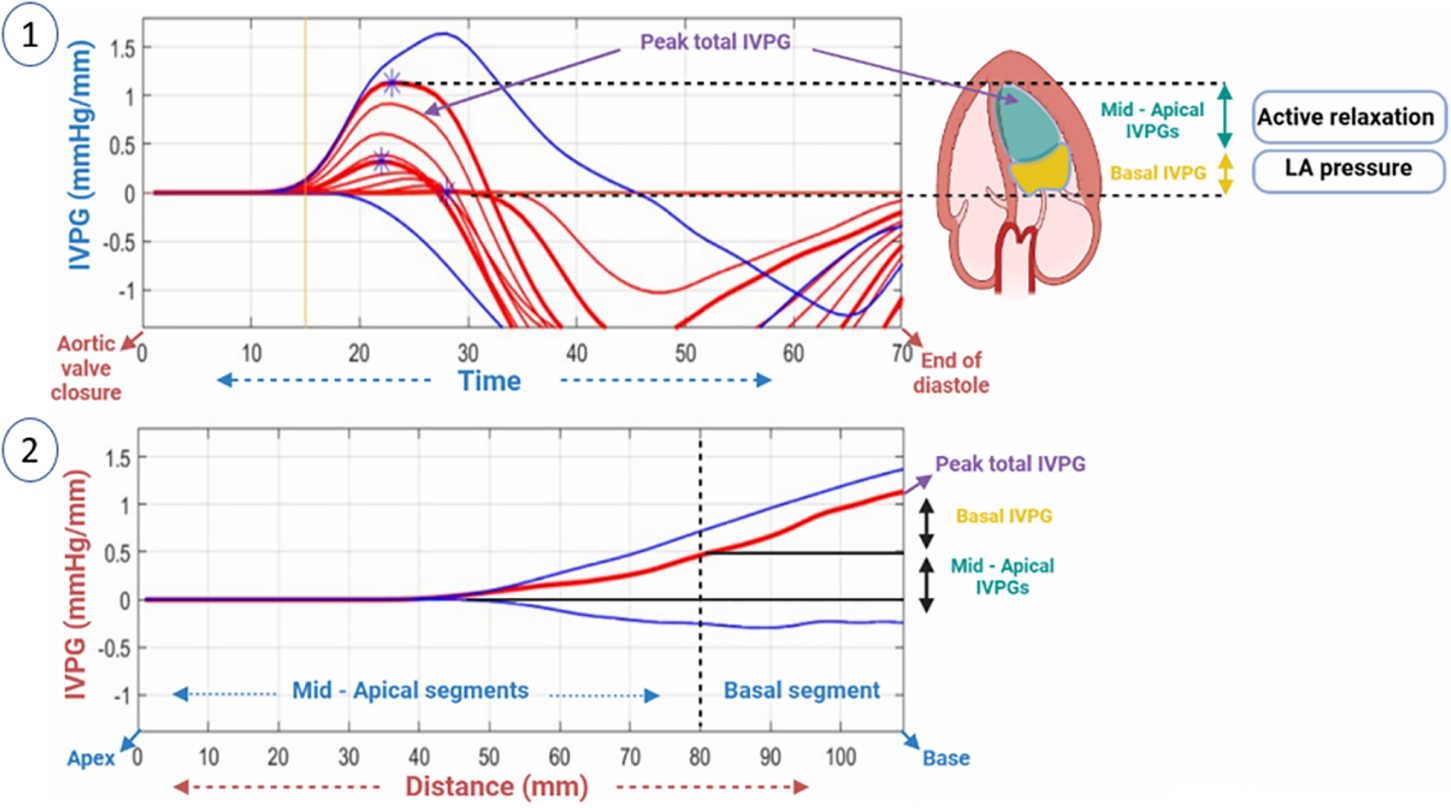
A two-dimensional graph of the temporal profile of the IVPG over a certain percentage of systole from the time of closure of the aortic valve to the end of diastole is displayed, highlighting the peak total IVPG and segmental IVPGs respectively (1). Additionally, a two-dimensional spatial profile of the IVPG from the apex to the base (MV) of the heart is presented, with the red line symbolizing the spatial profile at peak total IVPG which generally occurs at the level of the MV. The top and bottom blue lines signify the inertial and convective IVPGs, respectively. Determining the basal and mid-apical segmental IVPGs was based on the LV segmentation rule (one-third for basal and two-thirds for mid-apical) (2).

The symbols P, s, ρ, v, and t represent pressure, a specific point on the scan line, blood density, velocity of the transmitral flow, and time respectively. Utilizing de-aliasing, images were recreated based on these parameters. Velocity field reconstructions then enabled the computation of relative pressures in the specified area (34) as shown in Figures 1, 2.

For software analysis of pressure variations in the LV, it was divided into four segments: basal-IVPG located at the mitral valve, apical-IVPG situated at the apex, mid-IVPG found in the central segment, and mid-to-apical IVPG lying between the basal and apical segments. Total IVPG was used to calculate total pressure. As shown below, the intraventricular pressure gradient (IVPG) was calculated as follows (12, 35):

$$IVPG\;(mmHg/cm)=\frac{IVPD}{LV\;length}.$$

### 2.6. Electrocardiographic data acquisition

The ECG signals were recorded in three groups at the end of the experiment using needle electrodes connected to Power Lab hardware (ML880 Power Lab 16/30, AD Instruments) and Lab Chart Pro software (Lab Chart v8, AD Instruments) following a previously published protocol (36). For ECG measurement, the Power Lab was set up according to manufacturer instructions. Ten minutes after anesthesia, recording began and the most stable, uninterrupted section from each record was chosen for evaluation. Heart rate (HR), atrial complex (PR interval and *P* wave duration), and ventricular complexes (QRS complex and R amplitude) were recorded and analyzed.

### 2.7. Measurements of heart rate variability (HRV)

#### 2.7.1. Settings for data analysis

HRV analysis was performed on electrocardiographic recordings using Lab Chart 8 (ADInstruments) and the HRV module, as previously reported (37). The full 10 min recording was analyzed, using the company defaults for the rat provided by the Lab Chart HRV module. Ectopic beats were not included in the calculation. The software provided a report containing HRV parameters, which were then exported to Microsoft Excel for quantification (38).

#### 2.7.2. Parameters employed to describe heart rate dynamics

To evaluate HRV, time- and frequency-domain variables were utilized (39–41). Time-domain variables such as the SDRR, CVRR (calculated by dividing SDRR and average RR interval), and RMSSD (representing short-term variability) were used in the analysis. Frequency-domain variables included total power (ms^2^) across the entirety of the examined power spectrum (0–5 Hz). The RR interval oscillations were divided into three parts—very low-frequency power (0–0.15 Hz), low-frequency power (0.15–1.5 Hz), and high-frequency power (HF, 1.5–5 Hz) (37, 39, 42).

### 2.8. Gross evaluation and image scoring

Multiple images were captured at the time of euthanasia before cutting the hearts for gross evaluation of the severity and size of the lesion in the myocardium. Moreover, for further interpretation, we used the free ImageJ software for quantitative analysis of the gross score (ImageJ, MD, USA) as described previously (43). The mean gray value, integrated density (ID), and median were measured (44). The mean gray value represents the average pixel values within the selected area of the image based on a reverse scale of gray shades (1–255), where 1 corresponds to black and 255 corresponds to white. This was used to measure density, which represents the severity of inflammation. Integrated density was represented by the sum of the values of the pixels and the median value of the pixels in the image, evaluated by Median (45). The results from the image values were then used for analysis.

### 2.9. Histological examination

All animals were euthanized using the inhalation method of isoflurane overdose. After necropsy and macroscopic examination, the heart was dissected into small pieces and preserved in 10% neutral buffered formalin for fixation. The heart tissue was sectioned into 5 -µm slices using an automatic benchtop tissue processor (LEICA TP 1020, Biosystem Tokyo, Japan), deparaffinized, rehydrated, and stained with hematoxylin and eosin (H&E) to check for inflammatory changes in the cardiac tissue, and Masson's trichrome stain (MTC, Junsei Chemical, Tokyo, Japan) to determine any fibrosis in the heart. Ten sections were randomly selected from each group (Sham, MI, and MI + TRE) for further statistical analysis. The slides were examined under a light microscope at 10× and 40× magnification, and images were captured using image software (CellSens Standard; Olympus, Tokyo, Japan).

The histopathological score was previously quantified in detail by Shi et al. (46). Briefly, a blinded histopathologist analyzed ten sections per group for the presence of mononuclear cell infiltration, interstitial edema, necrosis, and myocyte arrangement (assembled or disassembled and direction of myocardial cells). The results for all sections in the three groups were graded on a scale of 0 (none), 1 (mild), 2 (moderate), 3 (severe), or 4 (very severe). Fibrosis intensity between cardiomyocytes (interstitial level) and perivascular fibrosis were evaluated for MTC.

### 2.10. Immunohistochemical staining of CD3

Formalin-fixed, paraffin-embedded sections were deparaffinized and rehydrated to assess the tissue's immunohistochemical changes. Prior to the examination, the cardiac tissues were treated with citrate buffer (pH 7.0) at 92°C for 20 min, followed by hydrogen peroxide in methanol. After that, the sections were incubated in a blocking buffer (10% goat serum, 3% skim milk, 0.2% Tween 20 in PBS) for two hours at room temperature. After that, the slides were incubated overnight at refrigerator temperature with an anti-CD3 antibody (SP7, ab1669, dilution 1:150, Abcam). The sections were subsequently incubated with HRP-conjugated goat anti-rabbit as a secondary antibody. Between each step, washing with PBS was performed 3–5 times, each for 5 min. The sections were then incubated with DAPI (Dako EnVision+ Kit/HRP; Agilent Technologies Ltd) to visualize the immunolabeled cells. Finally, the slides were counterstained with hematoxylin and examined under a light microscope (BX43F, Olympus; Tokyo, Japan). The number of positive CD3 cells per 1 mm^2^ in each section was counted using CellSens (Olympus Co.).

### 2.11. Quantitative real-time polymerase chain reaction

RNA was isolated from heart tissue and reverse transcribed to detect the expression of chosen genes through quantitative real-time polymerase chain reaction (qPCR). As mentioned earlier, RNA extraction, reverse transcription, and real-time PCR were conducted (47). Total RNA was extracted using the FastGene RNA Premium Kit following the manufacturer's instructions. RNA concentration and purity were analyzed via the NanoDrop 2000 ultra-micro spectrophotometer (Thermo Fisher Scientific). First-strand cDNA was synthesized with the PrimeScript RT reagent Kit (Takara Bio) in accordance with the instructions. A PCR reaction was carried out with THUNDERBIRD® Next SYBR® qPCR Mix (TOYOBO Life Science) utilizing primer sequences listed in Table 1 (47, 48). The thermocycling conditions for qPCR consisted of 95°C for 30 s, followed by 40 cycles of 95°C for 5 s and 60°C for 30 s. Relative quantification was computed and normalized to β-actin. Data is displayed in terms of levels relative to the expression level in the control cells (49).

**Table 1:** Primers used in qPCR.

Gene	Forward	Reverse
IL-1β	5′-TCAAGCAGAGCACAGACCTG-3′	5′-ACTGCCCATTCTCGACAAGG-3′
TNF-α	5′-TCTTCAAGGGACAAGGCTGC-3′	5′-CGGAGAGGAGGCTGACTTTC-3′
IL-18	5′-AGGACTGGCTGTGACCCTAT-3′	5′-TCCTGGCACACGTTTCTGAA-3′
Bcl-2	5′-CGACTTTGCAGAGATGTCCA-3′	5′-CATCCACAGAGCGATGTTGT-3′
BAX	5′-CAACATGGAGCTGCAGAGGA-3′	5′-CCGTCTGCAAACATGTCAGC-3′
SOD1	5′-TAACTGAAGGCGAGCATGGG-3′	5′-ATGCCTCTCTTCATCCGCTG-3′
SOD2	5′-AGTGACATTGTGCCTCTGGG-3′	5′-AGGCCCTGCATACTTTGTCC-3′
SOD3	5′-ACTTAAGCATCACCCAGGGC-3′	5′-ATTGAGGTGTCTGGGAAGCG-3′
β-actin	5′-CCCATCTATGAGGGTTACGC-3′	5′-TTTAATGTCACGCACGATTTC-3′

IL-1B, Interleukin 1 beta; TNF-α, tumor necrosis factor alpha; IL-18, interleukin-18; SOD1, CuZn-SOD; SOD2, Mn-SOD; SOD3, extracellular-SOD.

### 2.12. Statistical analysis

A prior estimate of the sample size based on one-way ANOVA was measured based on the outcomes and calculation performed with G*Power 3.1.9.2 software (50), with a 0.90 effect size (51). According to the results, the total sample size was 24 rats, divided into three groups, *n* = 8. All analyses were conducted using GraphPad Prism 8.0 (GraphPad Software, San Diego, California) and data were presented as mean ± standard deviation. The normality of the data was evaluated using the Shapiro–Wilk test. Statistical differences between groups (Sham, MI, and MI + TRE) were determined by repeated one-way ANOVA for nonparametric data using the Friedman test (as nonparametric tests are most useful for small studies) followed by Dunn's *post hoc* test. *Post hoc* testing was done using Dunn's Multiple Comparison Test. A *P*-value less than 0.05 was considered to indicate the significance of the data. Spearman's rank correlation and linear regression analysis were used to assess the relationship between HRV, IVPG, and conventional echocardiographic measurements. Coefficient of determination (*R*^2^) was computed from the sum of the squares of the distances of the points from the best-fit curve.

## 3. Results

### 3.1. Conventional echocardiography

The echocardiographic measurements to evaluate cardiac dimensions and performance are provided in Table 2. The LV dimensions (LVIDd and LVIDs) in the MI group showed a significant increase when compared to the Sham group (*P* = 0.017, 0.001 respectively). There was no significant change in the same parameters between the trehalose-treated group and the sham one.

**Table 2:** Assessment of cardiac structure and function using conventional echocardiography.

	Sham	MI	MI + TRE
IVSd (mm)	1.852 ± 0.154	1.532 ± 0.162*	1.808 ± 0.090
IVSs (mm)	3.104 ± 0.360	2.056 ± 0.192*	2.865 ± 0.138
LVIDd (mm)	7.076 ± 0.704	8.604 ± 0.667*	7.774 ± 1.090
LVIDs (mm)	3.656 ± 0.232	5.616 ± 0.202*	5.284 ± 0.258
LVPWd (mm)	2.260 ± 0.373	2.068 ± 0.140	2.230 ± 0.302
LVPWs (mm)	3.152 ± 0.355	2.388 ± 0.214*	3.055 ± 0.262^†^
EF%	88.20 ± 1.384	65.39 ± 1.982*	76.53 ± 0.852
FS%	51.28 ± 2.114	29.86 ± 1.302*	38.37 ± 0.745
eV	77.01 ± 2.855	62.64 ± 5.224*	73.26 ± 11.44
aV	37.73 ± 7.530	42.71 ± 5.066	39.48 ± 7.126
*E*/*A*	1.825 ± 0.212	1.478 ± 0.287*	1.937 ± 0.586
Sm	4.852 ± 0.650	3.540 ± 0.555*	4.147 ± 0.738
Em	6.750 ± 0.681	4.970 ± 0.503*	5.383 ± 0.359
Am	4.287 ± 0.399	4.663 ± 0.455	3.910 ± 0.544
*E*/Em	10.06 ± 0.933	14.00 ± 2.627*	13.49 ± 1.230‡
Em/Am	1.556 ± 0.145	1.163 ± 0.045*	1.402 ± 0.253

Data presented as mean ± SD (*n* = 8, for each group). IVSd and IVSs, interventricular septal thickness in end-diastole and systole, respectively; LVIDd and LVIDs, left ventricular diastolic and systolic internal diameter, respectively; LVPWd and LVPWs, left ventricular diastolic and systolic posterior wall thickness, respectively; EF, ejection fraction and FS, fractional shortening. eV, early diastolic transmitral flow velocity; aV, late diastolic transmitral flow velocity; *E*/*A*, early to late diastolic transmitral flow velocities ratio. Sm, left ventricular wall velocity at systole; Em, left ventricular wall velocity at early diastole; Am, left ventricular wall velocity at late diastole; Em/Am, early to the late diastolic velocity of the left ventricular wall; *E*/Em, early diastolic velocity mitral is to the early diastolic velocity of the LV wall ratio.

* Indicated the significance between sham and MI groups.

^†^ For the significance between MI and MI + TRE groups.

^‡^ For the significance between sham and MI + TRE groups.

On the other hand, there was a significant reduction in IVSd, IVSs, LVPWs, EF%, and FS% in the MI group compared to the sham group (*P* = 0.04, 0.003, 0.01, 0.0008, and 0.0008, respectively). However, no significant difference was seen between the same parameters of the MI + TRE and the sham groups. Additionally, the variations in LVPWd were not statistically significant among the groups.

The transmitral flow revealed that the early mitral velocity (*E*) significantly declined (*P* = 0.05) in the MI group compared to the sham one; however, their values did not significantly change between the MI + TRE and sham groups. Additionally, there was no significant difference in late mitral velocity (*A* wave) and *E*/*A* ratio among the different groups.

As for the TDI assessment, Sm, Em, and Em/Am ratios were significantly lower in the MI group in comparison to the sham group (*P* = 0.03, 0.008, 0.03 respectively). Moreover, the *E*/Em ratio was significantly higher in the MI and MI + TRE groups than in the sham group. There were no significant variations in Am among the groups. The TDI evaluations between the MI + TRE and the Sham groups were not significantly different except for the *E*/Em ratio. Besides, none of the reported parameters displayed a noteworthy change between the model group and the treated group apart from changes in LVPWs.

### 3.2. Color M-mode echocardiography

**Figure 3:**
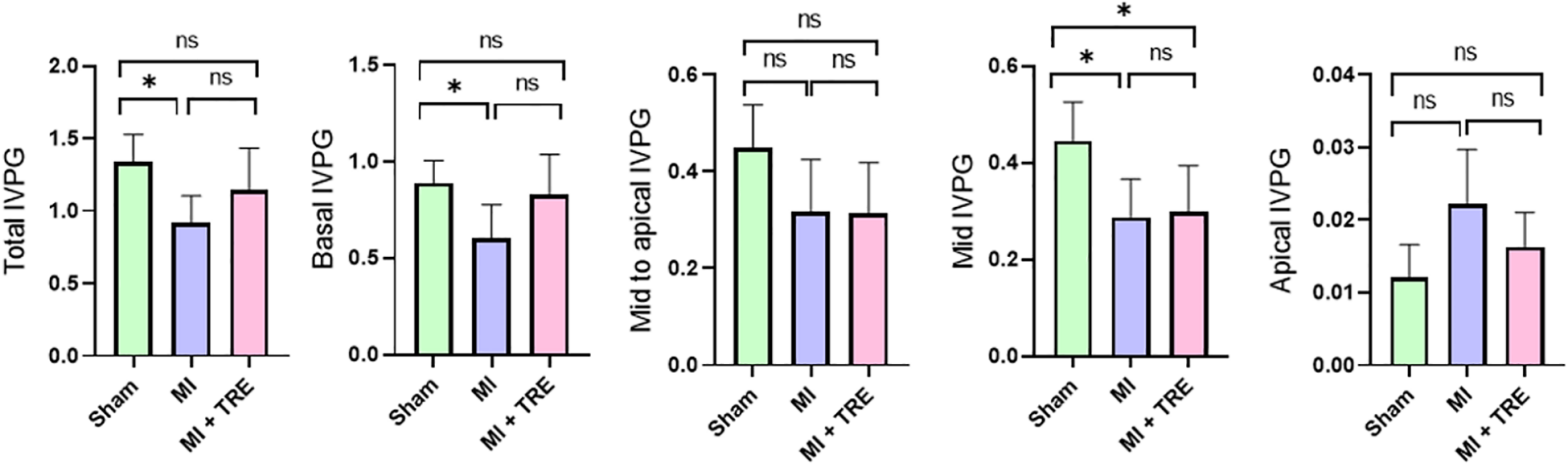
Bar charts showed the IVPG indices for the different groups, with the data represented as mean ± SD (*n* = 8, for each group). Mid to Apical IVPG and Apical IVPG exhibited no significant difference between the various groups. The Total IVPG, Basal IVPG, and Mid IVPG were significantly lower in the MI group than the Sham group. Trehalose seemed to improve these changes, presenting a non-significant distinction for the same parameters when compared to the Sham group apart from Mid IVPG (indicated by an asterisk for statistical significance, Ns *P* > 0.05, *P < 0.05).

The CMME evaluation of the LV flow is illustrated in Figure 3. The presence of myocardial infarction surgery influenced the IVPG; The MI group showed significantly smaller values for different IVPG parameters, including Total IVPG, Basal IVPG, and Mid IVPG, when compared to the sham group (*P* = 0.01, 0.03, and 0.02, respectively). On the other hand, in the trehalose-treated group, trehalose exhibited amelioration of the changes recorded in MI one, evidenced by a non-significant change with the sham group, except for Mid IVPG, which showed a significant decrease in the MI + TRE group compared to the sham one (*P* = 0.03). There was no notable difference in the Mid to Apical IVPG and Apical IVPG between the various groups.

### 3.3. ECG parameters

In ECG analysis, changes in heart rate, PR interval, P duration, QRS interval, and R amplitude are shown in Table 3. The MI group showed a significant increase in PR interval, P duration, and QRS interval, as well as a significant decrease in R amplitude compared with sham rats (*P* = 0.0110, 0.0112, 0.0442, 0.0029; respectively). There were no statistically significant variations between sham and MI + TRE for the same assessed variables (*P* = 0.99, 0.99, 0.75, 0.34; respectively). On the other hand, heart rate showed a significant reduction in MI compared to sham rats (136.5 ± 21.22 vs. 275.4 ± 10.08; *P* = 0.0001). Heart rate was enhanced after trehalose treatment but still significantly lower than sham rats (194.1 ± 25.00 vs. 275.4 ± 10.08; *P* < 0.0213).

**Table 3:** ECG parameters.

	Sham group	MI group	MI + TRE group
Heart rate (BPM)	275.4 ±** **10.08	136.5 **± **21.22*	194.1 **± **25.00^‡^
PR interval (s)	0.052 ±** **0.004	0.075 **± **0.005*	0.051 **± **0.007^†^
*P* duration (s)	0.020 ±** **0.004	0.034 **± **0.002*	0.021** ± **0.007^†^
QRS interval (s)	0.022 ±** **0.001	0.024 **± **0.0009*	0.015 **± **0.006^†^
*R* amplitude (mV)	0.940 ± 0.083	0.623 ± 0.156*	0.774 ± 0.286

Data are expressed as mean ± SD (*n* = 8, for each group). ANOVA, analysis of variance; MI, myocardial infarction, TRE, Trehalose.

* Indicated the significance between sham and MI groups.

† For the significance between MI and MI + TRE groups.

‡ For the significance between sham and MI + TRE groups.

### 3.4. Heart rate variability

**Figure 4:**
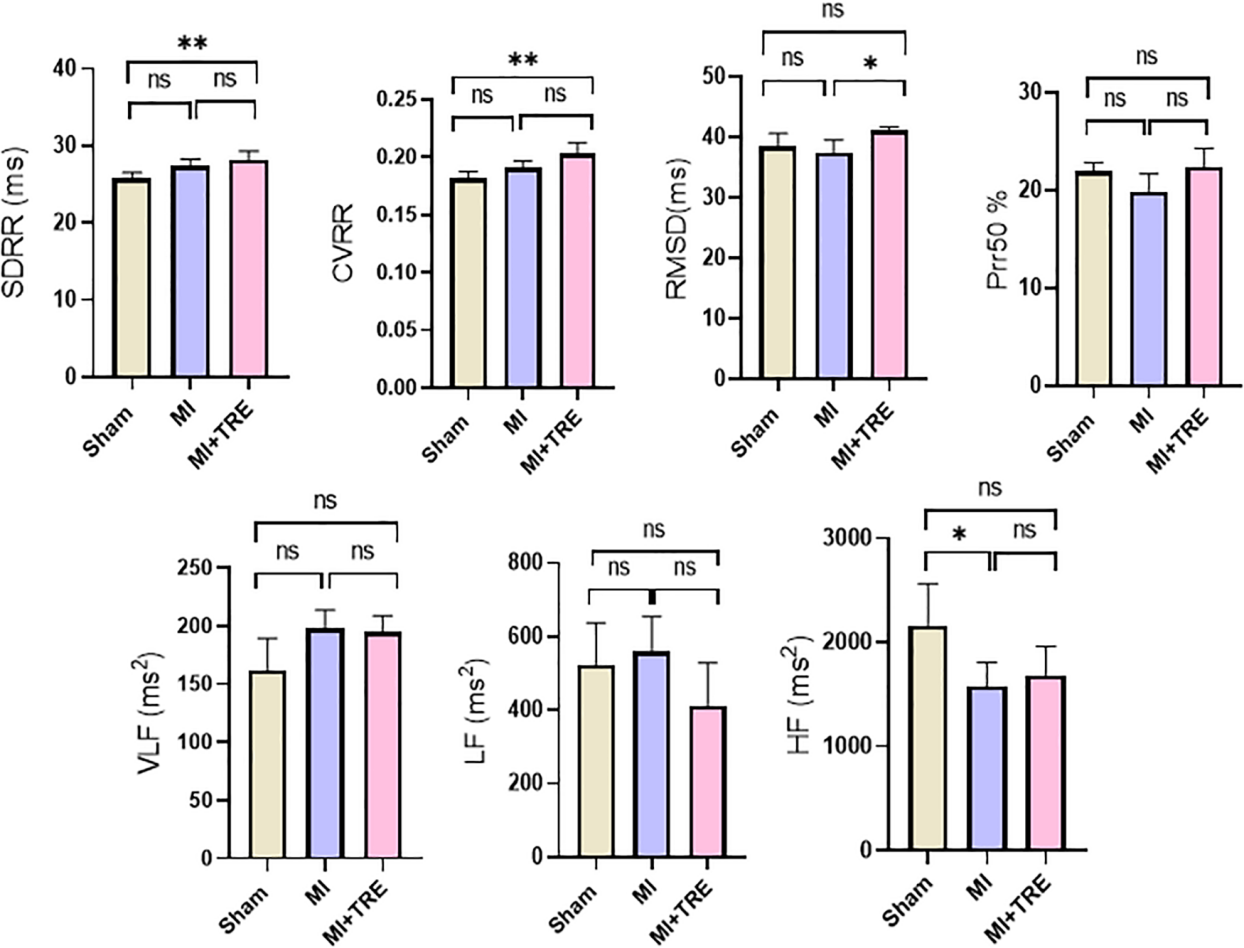
Bar graphs showed the heart rate variability parameters in the different groups represented by mean ± SD (*n* = 8, for each group). Abbreviations included standard deviation of RR intervals (SDRR), coefficient of variation of RR intervals (CVRR), square root of the mean of the squared differences between adjacent RR intervals (RMSSD), percentage of differences higher than 50 ms in RR intervals (pRR50), very low-frequency power (VLF), low-frequency power (LF), and high-frequency power (HF). The values for VLF, LF, and pRR50 showed no significant variations among the various study groups, while the HF was significantly lower in the MI group compared to the Sham one. The Trehalose-treated group demonstrated significantly higher SDRR and CVRR when compared to the Sham group, and a significantly higher RMSSD than the MI group (indicated by asterisk for statistical significance, Ns *P* > 0.05, *P < 0.05, **P < 0.01).

The analysis of HRV data (Figure 4) showed that there was no significant difference between the MI and MI + TRE groups compared to the sham group in RMSD (37.48 ± 2.113 and 41.09 ± 0.587 vs. 38.47 ± 2.164), pNN50% (19.88 ± 1.800 and 22.41 ± 1.869 vs. 21.95 ± 0.880), VLF (197.9 ± 15.95 and 194.7 ± 14.14 vs. 162.3 ± 27.23), and LF (560.6 ± 94.13 and 411.3 ± 117.0 vs. 524.4 ± 113.0). Only HF showed a significant decrease in the MI group compared to the sham group (1,578 ± 231.2 vs. 2,162 ± 402.5, *P* = 0.0374). Simultaneously, there was no significant alteration in the same parameter between the trehalose-treated and sham groups, highlighting the trehalose's potential to ameliorate this change in the treated rats. On the contrary, there was a significant increase in SDRR (28.13 ± 1.184 vs. 25.71 ± 0.821, *P* = 0.0042) and CVRR (0.202 ± 0.009 vs. 0.181 ± 0.005, *P* = 0.0020) in the trehalose-treated group compared to the sham group. Additionally, there was a significant increase in RMSD compared to the MI group (41.09 ± 0.587 vs. 37.48 ± 2.113, *P* = 0.01).

### 3.5. Effect of trehalose on the MI lesions

**Figure 5:**
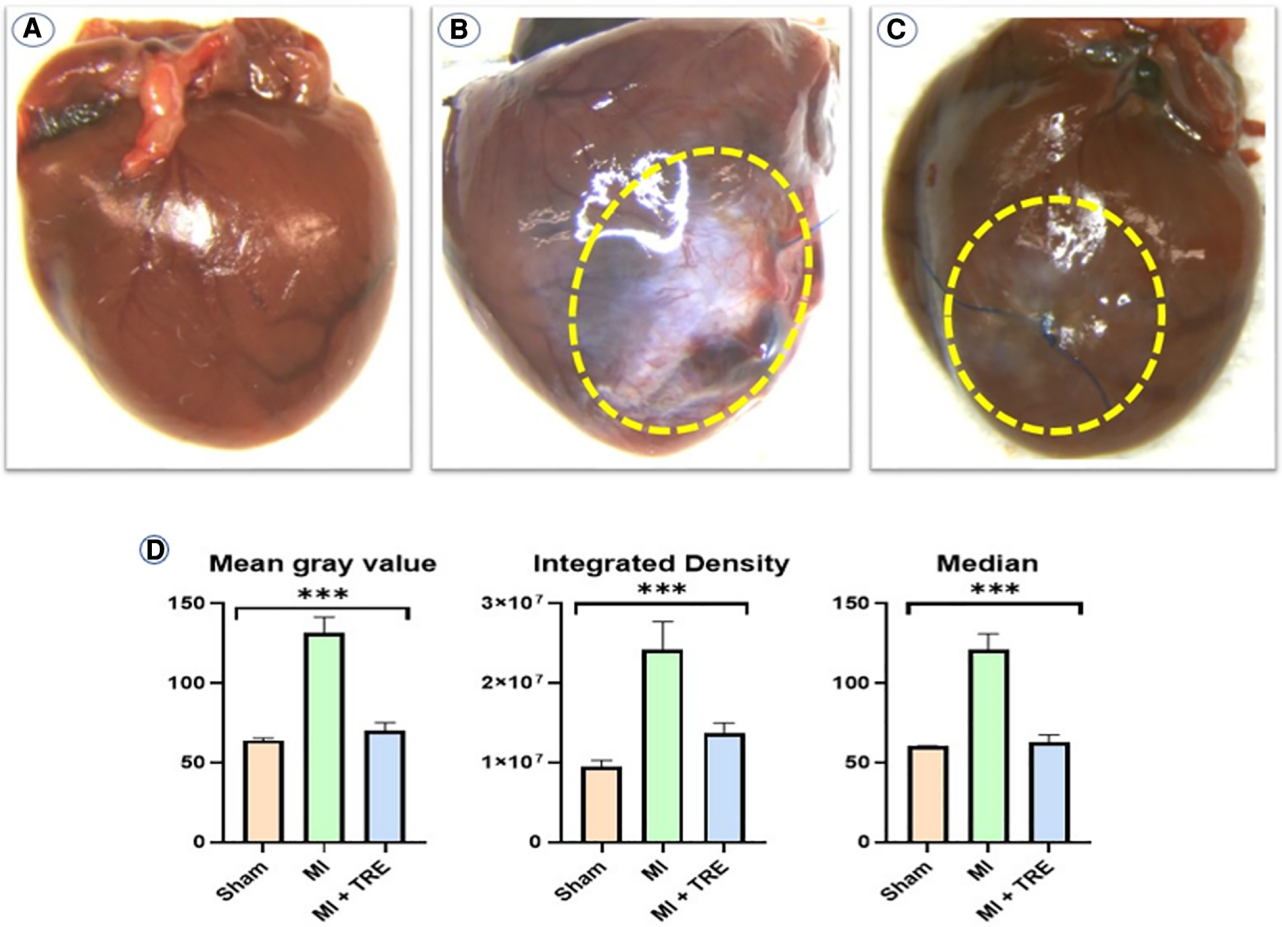
Gross examination of the myocardium in the three experimental groups revealed a significant difference in infarct size and density compared to each other: (**A**) the sham group had no myocardial lesion; (**B**) the MI group showed a characteristic, thickened epicardial lesion; and (**C**) the MI + TRE group had an obvious decrease in infarct size with resolution in the pathognomonic lesion as compared to the model group. (**D**) Analyzing data with Image J software, including Mean Gray Value, Integrated Density, and Median, among the three groups showed that the prescribed parameters were significantly higher compared to the Sham group. There was no significant difference in the measured values between the Sham and Treated (MI + TRE) groups (indicated by asterisk for statistical significance, ****P < 0.0001) (*n* = 8, for each group).

In the MI group, the myocardial lesion was significantly obvious and was characterized by thickened epicardium and patchy necrotic myocardial cells under the epicardium, accompanied by hyperplasia of granulation tissue or fibrous scar tissue (Figure 5B). Moreover, we observed an obvious decrease in infarct size with the resolution of the pathognomonic lesion in the MI + TRE group, which appeared more closely resembling the hearts of the sham group and unlike the MI group (Figures 5A,C). The results of the image quantification analysis of the hearts in the three groups are summarized in Figure 5D. In the MI group, the values of Mean gray value, integrated density (ID), and Median were significantly higher compared to the sham group (*P* = 0.0003). Conversely, there was no significant change in the measured values between the sham and treated (MI + TRE) groups (*P* = 0.15, 0.15, and 0.23, respectively).

### 3.6. Histopathological findings

**Figure 6:**
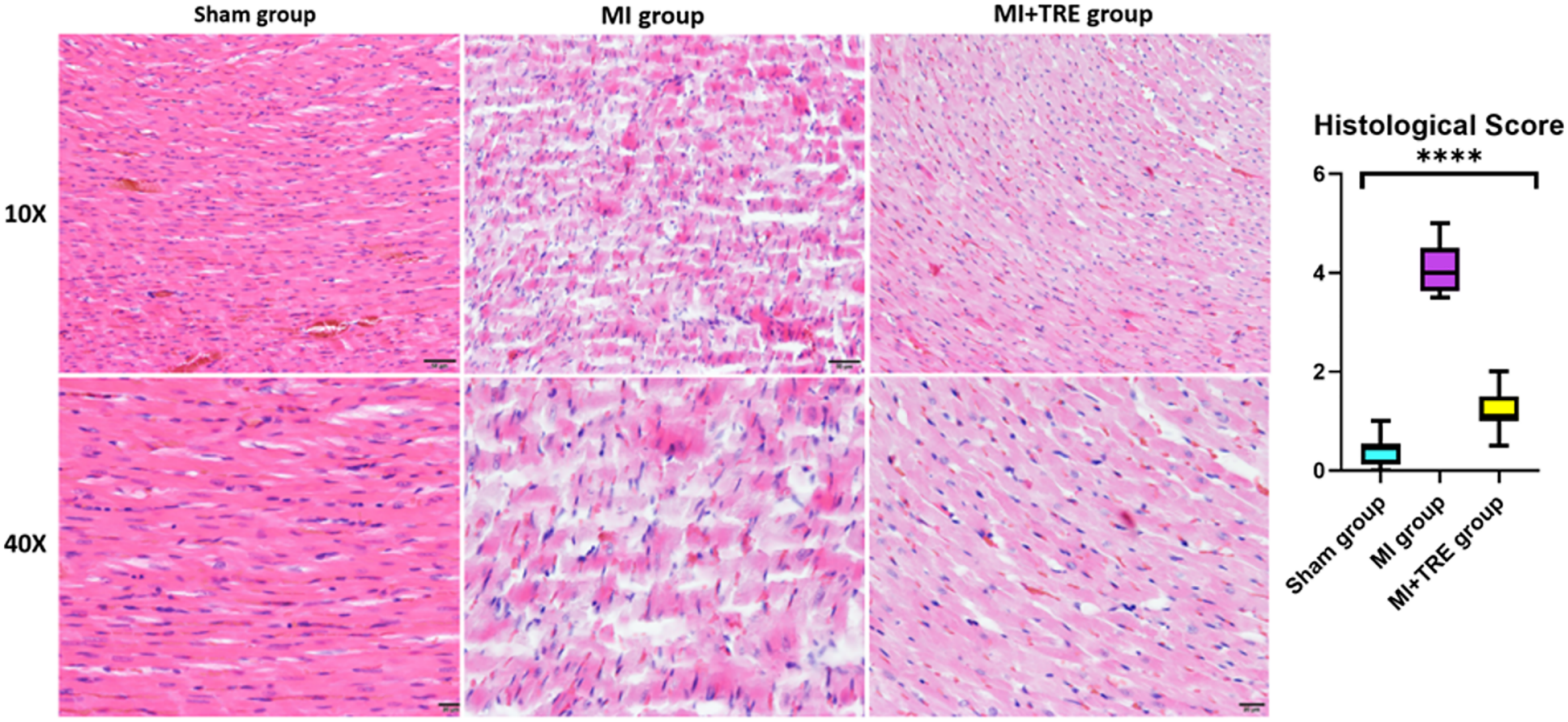
Histological examination of the heart tissue in the various groups observed at two levels of magnification (10× and 40×) indicated that the sham group had normal physical characteristics and maintained the structure of its tissue. The MI group, however, exhibited a high infiltration of inflammatory cells and intense ischemic necrotic tissue. Interstitial edema distorted cardiac muscle fibers, as well as disassembled cardiomyocytes with a wavy appearance, were also evident. The Trehalose-treated group showed that the overall inflammatory state was improved following the treatment with Trehalose, represented by decreased mononuclear cell extravasation and lack of edema. (J) The graph revealed the histopathological score between the three groups; the histopathological score was significantly lower in the Trehalose-treated group compared to the MI group (indicated by asterisk for statistical significance, ****P < 0.0001) (*n* = 8, for each group).

**Figure 7:**
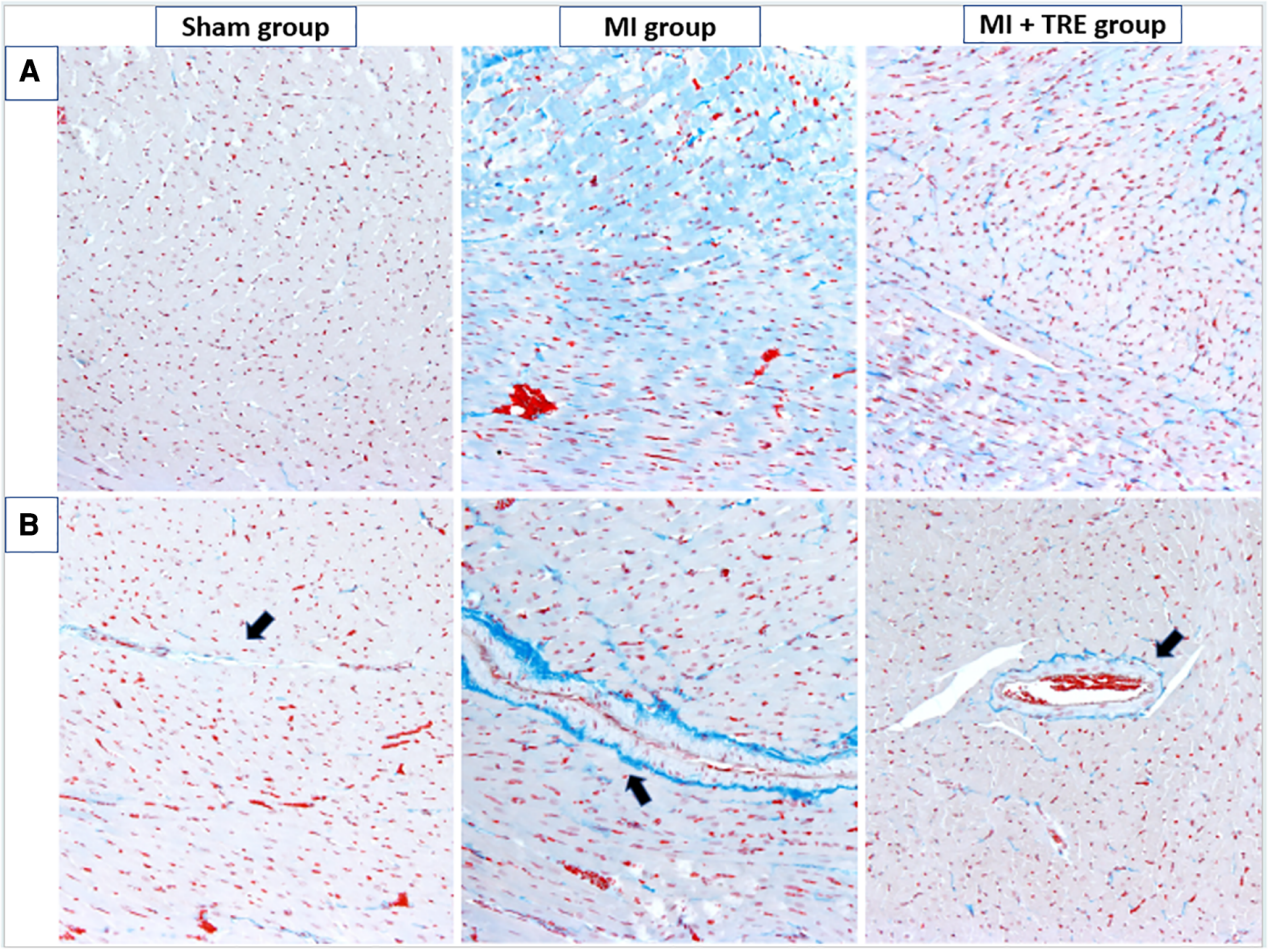
Cardiac tissue fibrosis evaluation. The severity of interstitial and perivascular fibrosis in the left ventricular myocardium was evaluated using Masson's trichrome staining on tissue sections, followed by examination under optical microscopy. The intensity of light blue staining indicates the extent of cardiac fibrosis. (**A**) shows peripheral myocardial interstitial fibrosis in the sham, MI, and MI + TRE groups, while (**B**) illustrates perivascular fibrosis in these three groups. After four weeks of acute MI, the MI group exhibited severe fibrosis at both interstitial and perivascular levels, in contrast to the treated group where moderate light blue staining was observed at both levels.

The non-treated sham heart appeared physically normal and preserved the tissue architecture when examined under the light microscope. The nuclei of cardiac cells were in the center and were large and eu chromatically stained. Minor areas of tissue damage from the trauma or handling of the heart during the experiment may be noticed. In contrast, the heart of the MI group showed a clear demarcation between the non-perfused and perfused parts of the myocardium. H&E staining revealed a high infiltration of inflammatory cells and severe ischemic necrotic tissue. Interstitial edema has been observed between the myocardial cells in response to the necrotic changes compared to the sham group. Distorted cardiac muscle fibers, as well as disassembled cardiomyocyte, were observed in the MI group compared to the sham and MI + TRE groups. In the MI + TRE group, the histopathological examination showed that the overall inflammatory status was improved after treatment with trehalose, represented by decreased mononuclear cell extravasation and absence of edema (Figure 6). The histopathological score between the three groups was significantly lower in the MI + TRE group compared to the MI group (*P* = 0.004), indicating that the administration of trehalose could decrease cardiac damage and pathological remodeling (Figure 6). Furthermore, The MI group showed a higher intensity of bright blue collagen staining compared to the other two groups, as seen in Figure 7, indicating the presence of fibrosis at both interstitial and perivascular levels. Notably, the intensity of fibrosis in the treated group was lower than in the sham group.

### 3.7. Immunohistochemical staining of CD3

**Figure 8:**
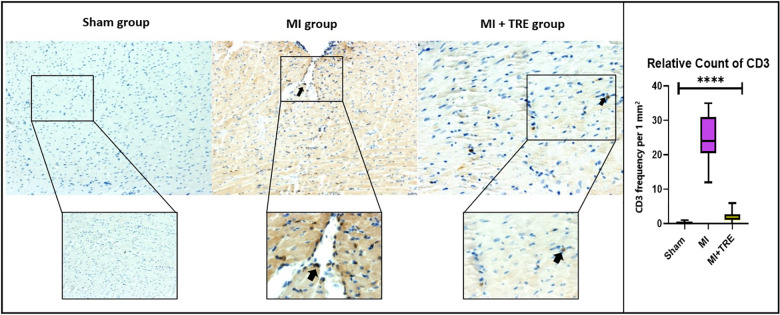
High-magnification photomicrographs illustrating immunohistochemistry of myocardial tissue following an infarction in rats. The images depict brown staining, indicating CD3-positive endothelial cells. The myocardium of the MI group exhibited a higher number of CD3+ cells, distinguished as brown particles under the microscope, compared to both the sham and MI + TRE groups. Quantitatively, the percentage of CD3+ cells in the MI group was significantly elevated four weeks post-surgery in comparison to the sham-operated and trehalose-treated rats. Statistical significance is denoted by an asterisk (****P < 0.0001).

To confirm the results of histopathological changes and study the type of inflammatory cell infiltration in the MI group compared to the sham and MI + TRE groups, the heart tissue was incubated with a monoclonal anti-CD3 antibody (a T cell inflammatory marker). We found that the number of CD3+ cells (represented as brown particles under the microscope) in the myocardium of the MI group was significantly higher than that in the myocardium of the sham and MI + TRE groups (*P* = 0.0001). On the contrary, there was no significant difference between the treated and sham groups (*P* = 0.07). Lowering the number of CD3+ cells in the treated group indicated that trehalose ameliorated the cardiac tissue injury induced by LAD ligation (Figure 8).

### 3.8. Real-time polymerase chain reaction

#### 3.8.1. Pro-inflammatory cytokines

**Figure 9:**
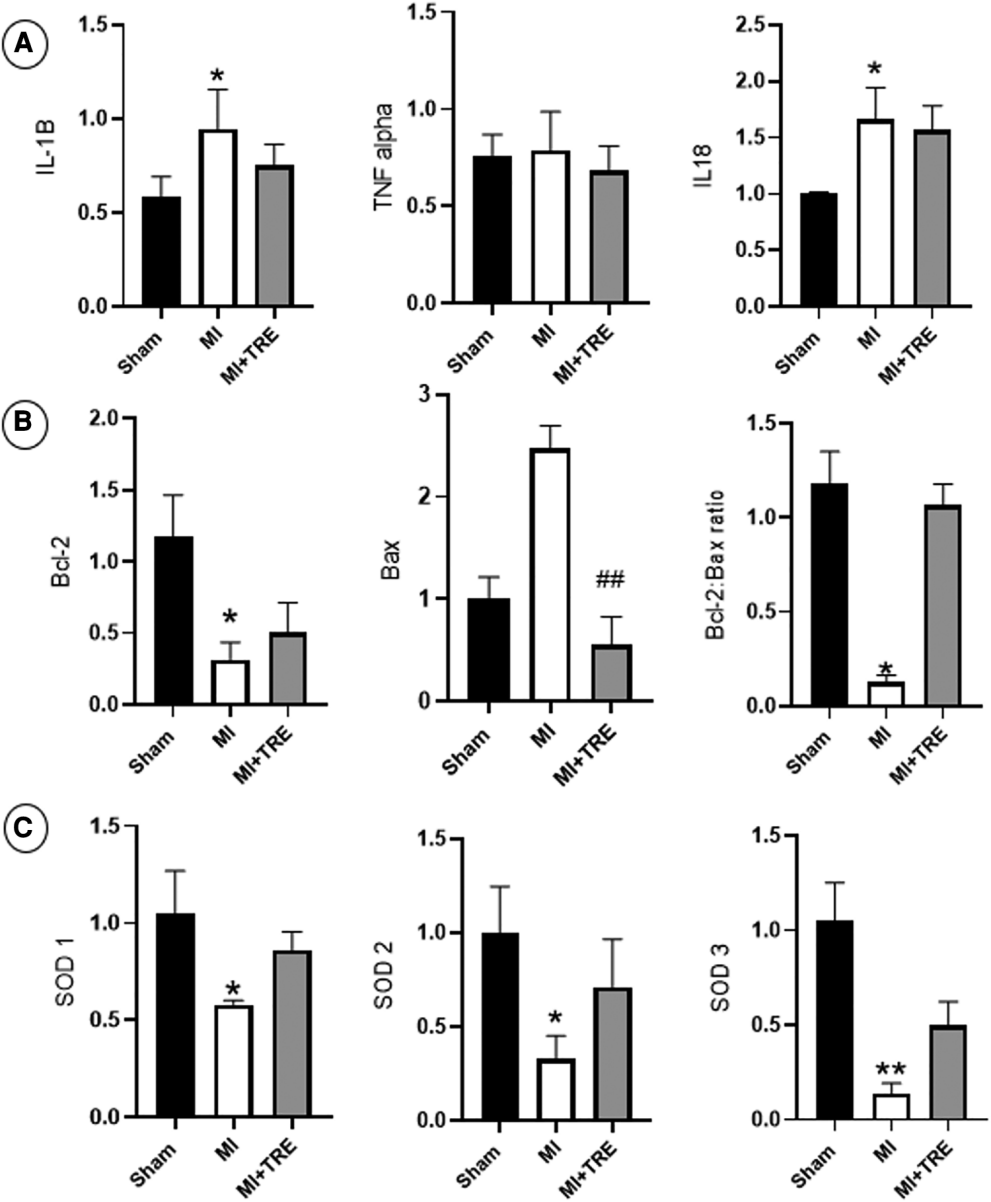
Bar graphs depicted the results of real-time PCR in the heart tissues from the study groups. (**A**) Levels of pro-inflammatory cytokines IL-1B and IL-18 increased significantly in the MI group compared to Sham rats. (**B**) In MI rats, The Bcl-2 family also exhibited a decrease in Bcl-2, an increase in Bax expression, and a decrease in the Bcl-2/Bax ratio relative to the sham group. (**C**) Superoxide dismutase isoenzymes SOD1, SOD2, and SOD3 had decreased expression levels in the MI group vs. those of the sham group, The asterisk is used to indicate the significance, Ns *P* > 0.05, *P < 0.05 (*n* = 8, for each group).

The levels of IL1-B and IL-18 increased significantly four weeks after surgery in the MI group compared to Sham rats (*P* = 0.032 and 0.02, respectively). However, there were no significant differences in the same measured values between the MI + TRE group and Sham group (*P* = 0.35 and 0.09, respectively). Additionally, the level of TNFα increased in the MI group compared to the other groups, though there was no significant change between them (Figure 9A).

#### 3.8.2. Bcl-2 family

We investigated the amounts of Bcl-2, Bax, and the ratio of Bcl-2 to Bax to explore if trehalose could modify the expression of the Bcl-2 family. In the MI group, the expression of Bcl-2 decreased significantly, while the expression of Bax increased, though not statistically significant, compared to the sham group (*P* = 0.013 and 0.28, respectively), and the Bcl-2/Bax ratio was significantly decreased (*P* = 0.018). In contrast, in the MI + TRE group, trehalose could ameliorate these changes by increasing Bcl-2 expression and significantly decreasing Bax expression (*P* = 0.007), leading to an increase in the Bcl-2/Bax ratio when compared to the MI group (Figure 9B).

#### 3.8.3. Superoxide dismutase isoenzymes SOD1, SOD2 and SOD3

The levels of SOD1, SOD2, and SOD3 were measured using quantitative real-time PCR to confirm the link between oxidative stress and impaired cardiac function. As shown in Figure 9C, in the MI group, the expression levels of SOD1, SOD2, and SOD3 were significantly reduced (*P* = 0.01, 0.01, and 0.005, respectively) compared with the sham group. Conversely, in the MI + TRE group, when given trehalose, the down-regulating tendencies of SOD1, SOD2, and SOD3 were noticeably reversed compared to the MI group, as evident by non-significant alterations in the expression levels of the specified genes compared to the sham group.

### 3.9. Correlation and regression analysis between IVPG indices and conventional echocardiographic parameters

Table 4 summarizes the correlation results between IVPG and conventional echocardiographic parameters and illustrates the coefficient of determination (*R*^2^) obtained from linear regression analysis, which indicates its effect on the same parameters.

**Table 4:** Correlation and regression analysis between IVPG and conventional echocardiographic parameters.

	Total IVPG		Basal IVPG		Mid to apical IVPG		Mid IVPG		Apical IVPG	
	*r*	*r* ^2^	*r*	*r* ^2^	*r*	*r* ^2^	*r*	*r* ^2^	*r*	*r* ^2^
IVSd (mm)	**0**.47*	0.19	**0**.49*	**0**.25*	0.25	0.02	0.31	0.06	−0.2	0.16
IVSs (mm)	**0**.6**	**0**.30*	**0**.59**	**0**.34*	0.39	0.09	**0**.53*	0.18	−0.3	**0**.24*
LVIDd (mm)	**−0**.72***	**0**.48**	**−0**.63**	**0**.44**	−0.44	**0**.25*	**−0**.57*	**0**.35**	0.13	0.1
LVIDs (mm)	**−0**.67**	**0**.33*	**−0**.51*	**0**.2*	**−0**.54*	**0**.34*	**−0**.68**	**0**.47**	0.13	0.1
LVPWd (mm)	−0.03	0.003	−0.08	0.002	0.03	0.002	−0.02	0.003	0.15	0.0007
LVPWs (mm)	**0**.53*	**0**.24*	**0**.49*	**0**.25*	0.33	0.09	0.38	0.13	0.11	0.05
EF%	**0**.71***	**0**.4**	**0**.64**	**0**.35**	**0**.49*	**0**.23*	**0**.67**	**0**.36**	−0.3	**0**.23*
FS%	**0**.7**	**0**.39**	**0**.64**	**0**.32*	**0**.47*	**0**.24*	**0**.66**	**0**.38**	−0.32	**0**.22*
eV	**0**.61**	**0**.44**	**0**.51*	**0**.38**	**0**.5*	**0**.26*	**0**.54*	**0**.32*	−0.04	0.02
aV	**−0**.68**	**0**.38**	**−0**.6**	**0**.36**	**−0**.54*	0.16	**−0**.57*	0.19	−0.21	0.005
*E*/*A*	**0**.69**	**0**.44**	**0**.57*	**0**.43**	**0**.54*	0.19	**0**.59**	**0**.2*	0.13	0.000001
Sm	0.32	0.09	0.21	0.06	0.26	0.07	0.37	0.15	−0.39	**0**.2*
Em	**0**.64**	**0**.34*	**0**.58*	**0**.22*	**0**.46*	**0**.32*	**0**.67**	**0**.47**	−0.38	0.17
Am	−0.3	0.06	−0.36	0.09	−0.06	0.005	−0.16	0.006	−0.09	0.0001
E/Em	**−0**.47*	0.17	−0.32	0.07	**−0**.56*	**0**.27*	**−0**.69**	**0**.35**	0.02	0.05
Em/Am	**0**.**64**	**0**.38**	**0**.7**	**0**.4**	0.33	0.13	**0**.54*	**0**.23*	−0.31	**0**.23*

*, **, *** Respectively represent significance *P* < .05, *P* < .001, *P* < .0001 and are shown in bold.

Both Total IVPG and Basal IVPG showed a significant positive correlation with IVSd, IVSs, LVPWs, EF%, eV, *E*/*A*, Em, and Em/Am, while a significant negative correlation was observed between these IVPG indices and LVIDd, LVIDs, and aV. Furthermore, a significant negative correlation was observed between Total IVPG and *E*/Em (*r* = −0.47, *P *= 0.04). Mid-to-apical IVPG and Mid-IVPG showed significant positive correlations with EF%, FS%, eV, *E*/*A*, and Em, while a significant negative correlation was observed between these IVPG indices and LVIDs, aV, and *E*/Em. Mid-IVPG displayed a significant positive correlation with IVSs and Em/Am (*r* = 0.53, *P *= 0.02, *r* = 0.54, *P *= 0.01 respectively), as well as a significant negative correlation with LVIDd (*r* = −0.57, *P *= 0.02). In contrast, no significant correlation was observed between Apical IVPG and these echocardiographic parameters.

A statistically significant effect of Total IVPG was observed on IVSs, LVIDd, LVIDs, LVPWs, EF%, FS%, eV, aV, *E*/*A*, Em, and Em/Am (*R*^2 ^= 0.30, 0.48, 0.33, 0.24, 0.40, 0.39, 0.44, 0.38, 0.44, 0.34, and 0.38, respectively).

Basal IVPG exhibited a significant effect on IVSd, IVSs, LVIDd, LVIDs, LVPWs, EF%, FS%, eV, aV, *E*/*A*, Em, and Em/Am (*R*^2 ^= 0.25, 0.34, 0.44, 0.20, 0.25, 0.35, 0.32, 0.38, 0.36, 0.43, 0.22, and 0.40, respectively). Mid-to-apical IVPG showed a significant effect on LVIDd, LVIDs, EF%, FS%, eV, Em, and *E*/Em (*R*^2 ^= 0.25, 0.34, 0.23, 0.24, 0.26, 0.32, and 0.27, respectively). Mid-IVPG also presented a significant effect on LVIDd, LVIDs, EF%, FS%, eV, *E*/*A*, Em, and *E*/Em (*R*^2 ^= 0.35, 0.47, 0.36, 0.38, 0.32, 0.20, 0.47, and 0.35, respectively). Finally, Apical IVPG demonstrated a significant effect on IVSs, EF%, FS%, and Sm (*R*^2 ^= 0.24, 0.23, 0.22, and 0.20, respectively).

### 3.10. Correlation and regression analysis between heart rate variability and conventional echocardiographic parameters

Table 5 summarizes the correlation results between heart rate variability and conventional echocardiographic parameters and their effect on the same parameters, illustrated by the coefficient of determination (*R*^2^) obtained from linear regression analysis.

**Table 5:** Correlation and regression analysis between heart rate variability and conventional echocardiographic parameters.

	SDRR (ms)		CVRR		RMSD (ms)		Prr 50%		VLF (ms^2^)		LF (ms^2^)		HF (ms^2^)	
	*r*	*r* ^2^	*r*	*r* ^2^	*r*	*r* ^2^	*r*	*r* ^2^	*r*	*r* ^2^	*r*	*r* ^2^	*r*	*r* ^2^
IVSd (mm)	−0.3	0.06	−0.23	0.008	0.21	0.08	**0**.63**	**0**.38**	−0.36	0.06	**−0**.52*	**0**.2*	0.23	0.09
IVSs (mm)	−0.39	0.06	−0.28	0.005	0.28	0.08	**0**.43*	**0**.23*	−0.43	0.07	−0.42	0.15	**0**.53*	**0**.29*
LVIDd (mm)	0.23	0.02	0.16	0.0001	−0.24	0.06	**−0**.56*	**0**.26*	0.11	0.002	0.01	0.01	**−0**.56*	**0**.34*
LVIDs (mm)	**0**.52*	**0**.44**	0.45	**0**.32*	−0.11	0.006	**−0**.52*	0.12	**0**.55*	**0**.28*	0.21	0.004	**−0**.57*	**0**.4**
LVPWd (mm)	−0.12	0.05	−0.08	0.03	0.25	0.12	0.17	0.04	−**0**.55*	0.08	−0.18	0.04	−0.01	0.006
LVPWs (mm)	−0.25	0.08	−0.15	0.009	**0**.55*	**0**.26*	**0**.59**	**0**.4**	−0.4	0.05	−0.24	0.04	0.15	0.12
EF%	**−0**.56*	**0**.27*	**−0**.47*	0.12	0.1	0.01	**0**.51*	**0**.2*	−0.43	0.14	−0.37	0.02	**0**.53*	**0**.38**
FS%	**−0**.55*	**0**.33*	**−0**.46*	0.17	0.1	0.003	**0**.49*	0.18	−0.4	0.18	−0.37	0.01	**0**.55*	**0**.39**
eV	−0.2	0.01	−0.15	0.0001	0.18	0.05	**0**.58*	**0**.37**	−0.09	0.003	−0.13	0.002	**0**.49*	**0**.27*
aV	0.07	0.01	0.02	0.0002	0.02	0.003	**−0**.53*	**0**.22*	0.07	0.01	0.5*	0.07	−0.36	0.01
*E*/*A*	0.008	0.01	0.05	0.06	0.02	0.03	**0**.51*	**0**.35**	0.03	0.02	−0.41	0.06	0.32	0.04
Sm	**−0**.45*	0.1	−0.42	0.07	−0.05	0.007	0.18	0.02	−0.15	0.0008	0.13	0.03	**0**.5*	**0**.28*
Em	**−0**.58*	**0**.27*	**−0**.51*	0.18	0.01	0.01	0.24	0.02	−0.26	0.13	−0.1	0.01	**0**.56*	**0**.41**
Am	−0.014	0.00002	−0.12	0.04	−0.45	0.17	−0.23	0.06	0.16	0.004	**0**.51*	**0**.25*	0.09	0.02
E/Em	**0**.69**	**0**.36**	**0**.69**	**0**.31*	0.2	0.08	−0.01	0.0003	0.45	0.18	−0.13	0.05	**−0**.49*	**0**.25*
Em/Am	−0.4	0.11	−0.28	0.02	0.2	0.02	0.34	0.12	−0.23	0.12	−0.34	0.07	0.38	0.18

*, **, *** Respectively represent significance *P* < .05, *P* < .001, *P* < .0001 and are shown in bold.

SDRR exhibited a significant positive correlation with LVIDs and *E*/Em, but also showed a significant negative correlation with EF%, FS%, Sm, and Em. Furthermore, CVRR demonstrated a significant negative correlation with EF%, FS%, and Em, while also displaying a significant positive correlation with *E*/Em. On the other hand, HF presented a significant positive correlation with IVSs, EF%, FS%, eV, Sm, and Em, as well as a significant negative correlation with LVIDd, LVIDs, and *E*/Em.

There was a significant effect of SDRR on LVIDs, EF%, FS%, Em, and *E*/Em (*R*^2 ^= 0.44, 0.27 0.33, 0.27, and 0.36, respectively). CVRR exhibited a significant effect on *E*/Em (*r*^2^ = 0.31 respectively). HF demonstrated a significant effect on IVSs, LVIDd, LVIDs, EF%, FS%, eV, Sm, Em, and *E*/Em (*R*^2^ = 0.29 0.34, 0.4, 0.38, 0.39, 0.27, 0.28, 0.41 and 0.25 respectively).

### 3.11. Correlation and regression analysis between IVPG indices and heart rate variability parameters

Table 6 summarizes the correlation results between IVPG and Heart Rate Variability parameters, as well as the effect of IVPG on the same parameters.

**Table 6:** Correlation and regression analysis between IVPG and heart rate variability parameters.

	Total IVPG		Basal IVPG		Mid to apical IVPG		Mid IVPG		Apical IVPG	
	*r*	*r* ^2^	*r*	*r* ^2^	*r*	*r* ^2^	*r*	*r* ^2^	*r*	*r* ^2^
SDRR (ms)	−0.2	0.03	−0.22	0.01	−0.21	0.06	−0.38	0.09	0.24	0.05
CVRR	−0.15	0.0004	−0.05	0.009	−0.27	0.05	−0.38	0.06	0.14	0.008
RMSD (ms)	0.22	0.09	0.41	**0**.2*	−0.18	0.003	−0.13	0.002	−0.03	0.002
Prr50%	**0**.7**	**0**.45**	**0**.76***	**0**.59***	0.28	0.07	0.34	0.1	−0.12	0.03
VLF (ms^2^)	−0.17	0.003	0.02	0.008	**−0**.51*	0.09	**−0**.49*	0.09	−0.21	0.009
LF (ms^2^)	−0.32	0.009	−0.38	0.05	−0.07	0.03	−0.12	0.02	0.11	0.05
HF (ms^2^)	**0**.54*	**0**.27*	**0**.48*	0.18	0.44	**0**.25*	**0**.57*	**0**.36**	−0.36	0.13

*, **, *** Respectively represent significance *P* < .05, *P* < .001, *P* < .0001 and are shown in bold.

Total IVPG displayed a significant positive correlation with Prr50% and HF. Basal IVPG was significantly positively correlated with the same HRV parameters. Mid-to-apical IVPG, and mid-IVPG, were significantly negatively correlated with the VLF parameter. Moreover, a significant positive correlation was observed between the mid-IVPG and HF (*r* = 0.57, *P *= 0.012).

There was a significant effect of Total IVPG on Prr50% and HF (*R*^2^ = 0.45 and 0.27 respectively). Basal IVPG demonstrated a significant effect on RMSD and Prr50% (*R*^2^ = 0.2 and 0.59 respectively). Mid to apical IVPG and Mid IVPG exhibited significant effects on HF (*R*^2^ = 0.25 and 0.36 respectively).

## 4. Discussion

It is widely known that myocardial infarction causes destructive remodeling of the left ventricle and a continuous decline in left ventricular systolic performance (52). Additionally, four to eight weeks after a major MI (≥ 40%) happens, rats start to show symptoms of traditional heart failure: diminished systolic ability (decreased ejection fraction and contractility), enhanced left ventricular end-diastolic pressure, and signs of volume overload (chest effusion, ascites, heightened lung weight, indicating pulmonary congestion). Therefore, at this period, it can be deemed systolic heart failure (52, 53). However, in the early phase after myocardial infarction, there is an evident left ventricular dysfunction, although there aren't any obvious signs of volume overload (54) as recorded in our study. Thus, our model can be described as acute post-MI LV systolic dysfunction.

After MI, the contractile and diastolic forces of the heart can significantly decline, particularly in the affected area. Reduced mobility due to ischemia in the ventricular walls is the cause of these alterations (55). Previous studies have demonstrated that the prognosis of myocardial infarction is not impacted by the presence or absence of symptoms, but instead depends on the severity of the lesion and the left ventricular function (56). Consequently, left ventricular function is essential for determining the efficacy of the treatment (55).

Echocardiographic assessment of cardiac function and remodeling is usually used as a qualification prerequisite for myocardial infarction. This technique continues to be a dependable, easily accessible, cost-efficient, and non-invasive inspection of cardiac performance in small animals (57). We found that trehalose treatment improves systolic function, as observed by improving LVIDs, EF, and FS%. Moreover, the diastolic function of the LV was also enhanced since trehalose effectively inhibits the increase of LVIDd and restores early mitral velocity. As a general observation of the conventional parameters, we can see that trehalose improves the systolic and diastolic function in infarcted rats compared to the MI group. Although the improvement was not significant in some parameters, we believe that longer administration of the medicine or an increase in the therapeutic dose may provide better results.

Color M-mode echocardiography can be utilized to assess diastolic function separately (58, 59). As in previous studies, software in MATLAB that was modified from earlier publications was used to calculate IVPG (11, 12). IVPG is a measure that reflects the suction force in the left ventricle (LV), which is observed by quick deformation of the LV wall followed by elastic recoil, released as stored elastic energy from the LV contraction during end-systole (60). A method for assessing heart function, IVPG allows for the differentiation of various pathological manifestations of diastolic dysfunction (12).

Our results showed that the total, basal, and mid-IVPG were reduced in the MI group and were approximately restored after trehalose administration. In contrast, a previous study revealed increased basal IVPG and decreased other segments, which can be attributed to the time course of MI (6 months vs. 1 month) and the severity of heart failure (33). In the early stages of HF, the total, basal, and mid-IVPG are reduced, which may be related to the reduced inflow (reduced E) and impaired recoil. In contrast, the advanced stage is characterized by an increased *E* wave. Basal IVPG is related to preloading, while mid-to-apical IVPG is related to elastic recoil. This suggests that IVPG is sensitive to early changes in HF before overt symptoms in our model. This reduction in total, basal, and mid-IVPG in the non-treated group due to impaired LV relaxation and cardiac fibrosis (infarct size%) is in line with a previous study (61). The results of the IVPG after trehalose treatment suggested that trehalose may improve elastic recoil by reducing the pathological score and remodeling the infarcted heart. As previously reported, trehalose significantly improved myocardial infarction in rat models (7).

Regardless of the non-significant difference between MI and MI treated groups, we found enhancement of the total and basal IVPG in Trehalose-treated group compared with the MI group. We think this may be due to the short time of treatment was able to enhance the cardiac remodeling but was not sufficient to provide significant change in the novel indices.

Electrocardiographic changes are often used to diagnose MI in humans and animals (62). In this study, we found that the ECG data of rats that had undergone LAD ligation in the MI group showed typical changes as in previous studies, which was accompanied by a significantly increased duration of the *P* wave, QRS complex, and PR duration. Additionally, the *R* wave was significantly reduced, which was consistent with the alterations noticed in the acute stage of anterior wall MI (myocardial infarction). This observation aligned with the outcomes of past studies (63–66).

On the other hand, operated groups presented a decrease in heart rate after four weeks, matching the outcomes of the previous study which explained this reduction through mechanistic and cellular activities involving cardiomyocyte Ca2+ handling. In a failing heart, a decrease in HR might be advantageous by granting greater time for SR Ca2+ stockpiling and more successful Ca2+ extraction from the cytoplasm, enhancing effective myocyte contraction and relaxation (67). Furthermore, in this study, we found that trehalose treatment for MI models alleviates the alterations in the electrophysiological characteristics of ischemic hearts vs. normal ones. Previous research revealed that the positive benefits of TRE might be mediated by the activation of the autophagic pathway (68–70).

The autonomous nervous system (ANS), which is composed of the sympathetic and parasympathetic systems, has a key role in cardiac electrophysiology (71). Furthermore, autonomic balancing regulates the electrical stability of cardiac cells with or without automatism, thus impacting the development of cardiac arrhythmias (72–74). An imbalance in the autonomic function of the heart after infarction has been documented in animal models (75, 76) and humans (77, 78).

Our results showed that no significant changes in most HRV parameters were observed after MI, including those in the time domain and frequency domain. These results are in agreement with those of Krüger and his colleagues (75). As previously mentioned, regarding HRV (heart rate variability) in rats, HRV parameters in the frequency domain possess a comparatively substantial interindividual variation, but not in the time domain (79). It appears that the assessment of Heart Rate Variability (HRV) in rats is not dependent on the absolute vagal tone, but rather the comparative impact between the vagal and sympathetic tone. Additionally, it has been found that the threshold for changes in HRV caused by a Myocardial Infarction (MI) may vary from other assessments of vagal activity. Lastly, following a MI, a decrease in HRV could be seen 56 days after the event in more severe cases of heart failure in rats (80).

In the frequency domain, we observed a significant decrease in HF. A weakened vagal tone has been associated with the diminished power of the HF band, while the LF band is thought to be influenced by both sympathetic and vagal tones (79, 81). HF symbolizes parasympathetic action, while LF stands for a fusion of both sympathetic and parasympathetic activities. Lower HF is connected to diminished parasympathetic activity (82). Signs of sympathetic activation have been frequently reported in patients during acute myocardial infarction (AMI) (83, 84). Lombardi and colleagues proposed that symptoms of sympathetic overactivity could still be observed in patients 14 days after AMI, regardless of the area of the infarct or their medication intake. Conversely, the high-frequency component declined, indicating a decrease in parasympathetic activity (85–87).

It has been well established that the relationship between abnormal HRV (decreased HRV), reduced autonomic nerve tone, and cardiovascular and cerebrovascular disorders are closely related (88). Previous research has demonstrated that patients with myocardial infarction exhibited structural alterations to the left ventricle as a result of a reduction in HRV (89). Additionally, decreased HRV was a strong predictor of all-cause mortality in patients with congestive heart failure (90). Also, reduced HRV may be associated with post-stroke morbidity and mortality (91). Furthermore, decreased HRV has been frequently reported in cerebrocardiovascular impairment and diabetes mellitus (92), insulin resistance (93), multiple sclerosis (94), muscular dystrophies (95), and Parkinson's disease (96). Therefore, HRV may be regarded as a therapeutic target (97).

As our study showed that Trehalose increased the SDRR, CVRR, and RMSD parameters of HRV and ameliorated HF. Additionally, we proposed that trehalose could reduce cardiac autonomic remodeling caused by MI. Nevertheless, further investigation is necessary to accurately determine the mechanisms of these results.

Our results demonstrated varying significant correlations and regressions among the IVPG, HRV, and conventional echocardiographic parameters. Specifically, our findings regarding IVPG were consistent with previously reported results in rats, dogs, and goats (33, 98, 99), which also showed varying correlations between heart function parameters and IVPG variables. Furthermore, we observed a significant correlation and regression between HRV measurement and heart function parameters (100, 101).

Regarding the relationship between HRV and IVPG, there was a significant correlation and regression observed between HRV and IVPG parameters. However, based on our current knowledge, this is the first study to explore the connection between these two novel techniques. Therefore, it is important to exercise caution when interpreting these relationships, as they may not be fully understood. Additionally, we aim to gather a greater number of studies in the future to enhance the statistical power of our findings.

Image analysis of heart tissue cannot differentiate between the causes of myocardial changes, but its measurement makes infarction scoring more reliable because it provides non-biased quantification of changes, away from the subjective or qualitative scoring that is commonly used in literature, in addition to increasing the statistical power (102). The beneficial effects of TRE treatment were associated with a reduction in MI lesions, which was evaluated by ImageJ software and evidenced by a decrease in the percentage of pixel intensity of the infarct area compared to the MI model (7).

Myocardial fibrosis is primarily caused by the proliferation of fibroblasts accompanied by imbalanced collagen deposition and degradation (103). While alternative fibrosis can prevent ventricular wall rupture (104), reactive interstitial fibrosis in the peripheral region may reduce ventricular compliance and increase stiffness, ultimately leading to a decline in cardiac function (105). Therefore, inhibiting cardiac fibroblast proliferation, collagen synthesis, and maintaining collagen homeostasis are crucial for preventing and reversing myocardial remodeling (106). Our study's findings were consistent with previous research (8), showing that Trehalose can alleviate myocardial fibrosis after MI by reducing interstitial and perivascular fibrosis. According to Garza and colleagues, cardiac remodeling after the onset of myocardial infarction (MI) is accompanied by structural changes in the left ventricle (LV), such as distorting tissue structure and increasing tissue stiffness, which account for ventricular dysfunction (107), They supported our results, which evaluated the structural changes that occurred in the ischemic hearts represented by the high level of inflammatory cells infiltration with severe ischemic necrotic tissue, interstitial edema, and distorted cardiac muscle fibers, as we declared the effect of trehalose on inhibition such as characteristic alteration in the wall structure of treated hearts. In addition, the difference between CD3-positive cells in MI, treated, and sham groups was significant (108), which indicate the effect of trehalose in eliminating the inflammatory reaction.

Inflammation is implicated in the pathology of myocardial fibrosis produced by hypertension, atherosclerosis, myocarditis, MI, and various diseases (109–112). MI is accompanied by the clearing of necrotic heart tissue and infiltration of inflammatory cells. Though inflammation is essential for repairing damaged tissue and spurring healing, ongoing inflammation can aggravate the damaging remodeling of cardiac tissue and cause heart failure (113). It is well known that the levels of pro-inflammatory cytokines are increased in patients with HF (114). In our study, MI heart was characterized by an increase in the level of IL-1β, IL-18, and TNFα (115), Trehalose decreased the elevation of the three cytokines. These results demonstrate that TRE attenuated the cardiac inflammation associated with MI. This can be related to the major pro-inflammatory cytokines that have general effects on macrophages, T lymphocytes, and cardiac myocytes (114).

During apoptosis, Bcl-2 proteins are pivotal, and Bax has a dominant role in inducing cell death by “making holes” in the mitochondrial membrane, thus disrupting its integrity and intensifying the emission of cytochrome C from mitochondria (116).

Generally, the up-regulation and down-regulation of members of the Bax and Bcl-2 family proteins can define whether cells survive or die by apoptosis in pathophysiology. Through our study, we found that in MI rats, Bax expression increased while Bcl-2 levels and the ratio of Bcl-2 to Bax decreased, which was linked to augmented apoptosis, comparable to former findings (116, 117). In contrast to the MI group, the aforementioned abnormal expressions of Bcl-2 and Bax, as well as the ratio of Bcl-2 to Bax, were reversed after TRE treatment. As a result, these findings suggest that TRE corrects abnormal expressions of Bcl-2 and Bax, augments the Bcl-2/Bax ratio, and blocks the initiation of mitochondrial apoptosis signaling.

SOD is a crucial enzyme; increasing its activity has been shown to benefit the cellular ability to scavenge/quench free radicals (118). The family of antioxidant enzymes known as SOD, which includes SOD1, SOD2, and SOD3, appears to be the initial and most effective line of defense against reactive oxygen species (ROS), particularly superoxide anion radicals (O2−). Experiment results showed that the expression levels of SOD1, SOD2, and SOD3 were much lower in the MI group, in agreement with a previous study (119). These down-regulated tendencies were efficiently reversed when treated with TRE, supporting our hypothesis.

## 5. Limitations

We did not compare TRE to the standard of care (reperfusion therapy +/− pharmacotherapies) or other glucose-mediated therapies. However, our focus was on investigating trehalose as a potential valuable pharmacological treatment for reducing cardiac remodeling and heart failure. While the comparison with other therapies can be addressed in future studies, we aimed to highlight the potential benefits of trehalose in this specific context.

Furthermore, plasma samples were not collected from the experimental rats during the study, as our primary focus was to evaluate the feasibility of using IVPG and HRV to detect changes in heart function in myocardial infarction (MI) rats treated with trehalose, rather than conducting extensive molecular analyses. However, it is worth mentioning that the potential combinations of these techniques with further molecular analysis could be explored in future studies.

## 6. Conclusion

The observed enhancements in conventional echocardiography, as well as the histological and immunohistochemical analyses, signify the positive influence of trehalose on the myocardial infarction rat model. These findings serve as a basis for future investigations aiming to investigate the therapeutic effects of trehalose and validate its potential benefits.

Although further research and investigation are necessary to fully ascertain the therapeutic impact of trehalose, our study offers valuable evidence of the correlation between IVPG and HRV with other established markers of echo assessment in the myocardial infarction rat model. These findings pave the way for future studies that could lead to the development of new treatments and improved outcomes for patients with myocardial infarction.

## Data Availability

The original contributions presented in the study are included in the article, further inquiries can be directed to the corresponding author.
